# Applying the compound Poisson process model to the reporting of injury-related mortality rates

**DOI:** 10.1186/1742-5573-4-1

**Published:** 2007-02-16

**Authors:** Scott R Kegler

**Affiliations:** 1 Office of Statistics and Programming, National Center for Injury Prevention and Control, Centers for Disease Control and Prevention, Atlanta GA, USA

## Abstract

Injury-related mortality rate estimates are often analyzed under the assumption that case counts follow a Poisson distribution. Certain types of injury incidents occasionally involve multiple fatalities, however, resulting in dependencies between cases that are not reflected in the simple Poisson model and which can affect even basic statistical analyses. This paper explores the compound Poisson process model as an alternative, emphasizing adjustments to some commonly used interval estimators for population-based rates and rate ratios. The adjusted estimators involve relatively simple closed-form computations, which in the absence of multiple-case incidents reduce to familiar estimators based on the simpler Poisson model. Summary data from the National Violent Death Reporting System are referenced in several examples demonstrating application of the proposed methodology.

## Introduction

Injury-related mortality rate estimates are often analyzed under the assumption that case counts follow a Poisson distribution. [1-4] Certain types of injury incidents occasionally involve multiple fatalities, however, resulting in dependencies between cases that are not reflected in the simple Poisson model and which can affect even elementary analyses of rates. This paper examines the application of the compound Poisson process model [5-7] to address this issue, emphasizing adjustments to some commonly used interval estimators for rates and rate ratios. Accompanying examples demonstrate the proposed adjustments and provide comparisons of results obtained under the Poisson and compound Poisson process models.

This paper was motivated by the need for basic statistical methods applicable to data from the National Violent Death Reporting System (NVDRS).[8] The NVDRS data provide a census of violent deaths occurring in the states covered by the reporting system. Data are collected on incidents and persons (victims/suspects), and records for all persons associated with each incident are linked. Two types of incidents (not mutually exclusive) are of particular note in the present context: (i) those involving multiple homicides and (ii) those involving homicide followed by suicide. The NVDRS data for the year 2004 (covering 13 states) indicate that over 4% of homicide-related incidents involved multiple homicides. Correspondingly, at the individual (person) level approximately 9% of homicides were associated with multiple-homicide incidents. These data also show that nearly 12% of homicide-suicide incidents involved multiple (usually two) homicides, while at the individual level approximately 23% of homicides associated with a homicide-suicide incident were part of a multiple homicide-suicide incident.

## Analysis

### An Analysis Framework Based on the Compound Poisson Process Model

Analyses of vital statistics data often rely on a conceptual framework in which case counts, even though based on a census, are considered inherently variable.[1,2,4,9,10] In selected National Center for Health Statistics reports, for example, mortality rate estimates are evaluated for statistical stability by assuming that census-level case counts (rate numerators) follow a Poisson distribution while at-risk population estimates (rate denominators) are assumed constant.[1,2]

The simple Poisson model includes the assumption that cases occur independently. Cases (fatalities) associated with multiple-case incidents are not independent, however, and for this reason such a model does not adequately characterize the types of incidents described above. The compound Poisson process model [5-7] provides a closer conceptual parallel, by incorporating a two-level counting process. Applying this model to the NVDRS data, incident counts represent the first level and are assumed to follow a simple Poisson distribution. The counts of cases associated with each incident represent the second level. These incident-specific case counts are assumed to follow a common (discrete) probability distribution of unspecified form. Incident-specific case counts are also assumed to be (i) independent across incidents and (ii) independent of the count of incidents. [5-7]

As an illustration of the basic aspects of the compound Poisson process model, suppose that occurrences of a specific type of incident are consistent with a Poisson process having person-year rate parameter λ. Letting person-years at risk be denoted by P, it follows that the incident count N has a Poisson distribution with (unknown) mean λP, denoted by N ~ Poisson(λP). At the next level, suppose that the incident-specific case counts C_1_, C_2_, C_3_, ..., C_N _have a common underlying distribution with mean μ and variance σ^2 ^(both generally unknown) and satisfy the independence assumptions specified above. The total case count C≡∑k=1NCk
 MathType@MTEF@5@5@+=feaafiart1ev1aaatCvAUfKttLearuWrP9MDH5MBPbIqV92AaeXatLxBI9gBaebbnrfifHhDYfgasaacH8akY=wiFfYdH8Gipec8Eeeu0xXdbba9frFj0=OqFfea0dXdd9vqai=hGuQ8kuc9pgc9s8qqaq=dirpe0xb9q8qiLsFr0=vr0=vr0dc8meaabaqaciaacaGaaeqabaqabeGadaaakeaacqqGdbWqcqGHHjIUdaaeWaqaaiabboeadnaaBaaaleaacqqGRbWAaeqaaaqaaiabbUgaRjabg2da9iabigdaXaqaaiabb6eaobqdcqGHris5aaaa@3885@ then conforms to a compound Poisson process model, with underlying mean E [C] = λP × μ and underlying variance Var(C) = λP × (μ^2 ^+ σ^2^). [5-7]

Effectively, the compound Poisson process model reduces to the simple Poisson model in analyses where multiple-case incidents do not occur.[7] In such situations C_k _= 1 for every incident, so that the total case count C is equal to the incident count N, with the latter variable assumed to follow a simple Poisson distribution. In this way, the framework based on the compound Poisson process model encompasses the more customary analysis framework based on the simple Poisson model.

### Rate Estimators and Variances

Using terms defined above, the typical estimate of the population-based case rate per 100,000 person-years is provided by R ≡ C/P × 100,000. Under the compound Poisson process model E [R] = E [C]/P × 100,000 = λ × μ × 100,000. Since this latter quantity also corresponds to the underlying case rate per 100,000 person-years, it follows that R is an unbiased estimator.

The variance of the rate estimator is Var(R) = Var(C)/P^2 ^× 100,000^2^. An unbiased estimate of Var(C) under the compound Poisson process model is conveniently provided by Va^r(C)=∑k=1NCk2
 MathType@MTEF@5@5@+=feaafiart1ev1aaatCvAUfKttLearuWrP9MDH5MBPbIqV92AaeXatLxBI9gBaebbnrfifHhDYfgasaacH8akY=wiFfYdH8Gipec8Eeeu0xXdbba9frFj0=OqFfea0dXdd9vqai=hGuQ8kuc9pgc9s8qqaq=dirpe0xb9q8qiLsFr0=vr0=vr0dc8meaabaqaciaacaGaaeqabaqabeGadaaakeaacqqGwbGvcuqGHbqygaqcaiabbkhaYjabcIcaOiabboeadjabcMcaPiabg2da9maaqadabaGaee4qam0aa0baaSqaaiabbUgaRbqaaiabikdaYaaaaeaacqqGRbWAcqGH9aqpcqaIXaqmaeaacqqGobGta0GaeyyeIuoaaaa@3E5E@ (see Appendix A). Substituting ∑k=1NCk2
 MathType@MTEF@5@5@+=feaafiart1ev1aaatCvAUfKttLearuWrP9MDH5MBPbIqV92AaeXatLxBI9gBaebbnrfifHhDYfgasaacH8akY=wiFfYdH8Gipec8Eeeu0xXdbba9frFj0=OqFfea0dXdd9vqai=hGuQ8kuc9pgc9s8qqaq=dirpe0xb9q8qiLsFr0=vr0=vr0dc8meaabaqaciaacaGaaeqabaqabeGadaaakeaadaaeWaqaaiabboeadnaaDaaaleaacqqGRbWAaeaacqaIYaGmaaaabaGaee4AaSMaeyypa0JaeGymaedabaGaeeOta4eaniabggHiLdaaaa@36A2@ in place of Var(C) provides the unbiased variance estimate Va^r(R)=∑k=1NCk2
 MathType@MTEF@5@5@+=feaafiart1ev1aaatCvAUfKttLearuWrP9MDH5MBPbIqV92AaeXatLxBI9gBaebbnrfifHhDYfgasaacH8akY=wiFfYdH8Gipec8Eeeu0xXdbba9frFj0=OqFfea0dXdd9vqai=hGuQ8kuc9pgc9s8qqaq=dirpe0xb9q8qiLsFr0=vr0=vr0dc8meaabaqaciaacaGaaeqabaqabeGadaaakeaacqqGwbGvcuqGHbqygaqcaiabbkhaYjabcIcaOiabbkfasjabcMcaPiabg2da9maaqadabaGaee4qam0aa0baaSqaaiabbUgaRbqaaiabikdaYaaaaeaacqqGRbWAcqGH9aqpcqaIXaqmaeaacqqGobGta0GaeyyeIuoaaaa@3E7C@/P^2 ^× 100,000^2^.

### Confidence Intervals for Rates

Confidence intervals for rates and rate ratios frequently involve an initial logarithmic transformation to the point estimate. Applying this transformation to the rate estimator R, the "delta" method [11-14] suggests the following general form (not model-dependent) for an approximate 95% confidence interval for the underlying rate [15]:

(exp⁡{ln⁡(R)−1.96×Va^r(R)R2},  exp⁡{ln⁡(R)+1.96×Va^r(R)R2}).     (1)
 MathType@MTEF@5@5@+=feaafiart1ev1aaatCvAUfKttLearuWrP9MDH5MBPbIqV92AaeXatLxBI9gBaebbnrfifHhDYfgasaacH8akY=wiFfYdH8Gipec8Eeeu0xXdbba9frFj0=OqFfea0dXdd9vqai=hGuQ8kuc9pgc9s8qqaq=dirpe0xb9q8qiLsFr0=vr0=vr0dc8meaabaqaciaacaGaaeqabaqabeGadaaakeaadaqadaqaaiGbcwgaLjabcIha4jabcchaWnaacmqabaGagiiBaWMaeiOBa4MaeiikaGIaeeOuaiLaeiykaKIaeyOeI0IaeGymaeJaeiOla4IaeGyoaKJaeGOnayJaey41aq7aaOaaaeaadaWcaaqaaiabbAfawjqbbggaHzaajaGaeeOCaiNaeiikaGIaeeOuaiLaeiykaKcabaGaeeOuai1aaWbaaSqabeaacqaIYaGmaaaaaaqabaaakiaawUhacaGL9baacqGGSaalcqqGGaaicqqGGaaicyGGLbqzcqGG4baEcqGGWbaCdaGadeqaaiGbcYgaSjabc6gaUjabcIcaOiabbkfasjabcMcaPiabgUcaRiabigdaXiabc6caUiabiMda5iabiAda2iabgEna0oaakaaabaWaaSaaaeaacqqGwbGvcuqGHbqygaqcaiabbkhaYjabcIcaOiabbkfasjabcMcaPaqaaiabbkfasnaaCaaaleqabaGaeGOmaidaaaaaaeqaaaGccaGL7bGaayzFaaaacaGLOaGaayzkaaGaeiOla4IaaCzcaiaaxMaadaqadaqaaiabigdaXaGaayjkaiaawMcaaaaa@6D7F@

When R is based on a total case count C that is assumed to follow a simple Poisson distribution, interval (1) simplifies to the more recognizable form [10,16,17]:

(exp⁡{ln⁡(R)−1.96×1C},  exp⁡{ln⁡(R)+1.96×1C}).     (1a)
 MathType@MTEF@5@5@+=feaafiart1ev1aaatCvAUfKttLearuWrP9MDH5MBPbIqV92AaeXatLxBI9gBaebbnrfifHhDYfgasaacH8akY=wiFfYdH8Gipec8Eeeu0xXdbba9frFj0=OqFfea0dXdd9vqai=hGuQ8kuc9pgc9s8qqaq=dirpe0xb9q8qiLsFr0=vr0=vr0dc8meaabaqaciaacaGaaeqabaqabeGadaaakeaadaqadaqaaiGbcwgaLjabcIha4jabcchaWnaacmqabaGagiiBaWMaeiOBa4MaeiikaGIaeeOuaiLaeiykaKIaeyOeI0IaeGymaeJaeiOla4IaeGyoaKJaeGOnayJaey41aq7aaOaaaeaadaWcaaqaaiabigdaXaqaaiabboeadbaaaSqabaaakiaawUhacaGL9baacqGGSaalcqqGGaaicqqGGaaicyGGLbqzcqGG4baEcqGGWbaCdaGadeqaaiGbcYgaSjabc6gaUjabcIcaOiabbkfasjabcMcaPiabgUcaRiabigdaXiabc6caUiabiMda5iabiAda2iabgEna0oaakaaabaWaaSaaaeaacqaIXaqmaeaacqqGdbWqaaaaleqaaaGccaGL7bGaayzFaaaacaGLOaGaayzkaaGaeiOla4IaaCzcaiaaxMaadaqadaqaaiabigdaXiabbggaHbGaayjkaiaawMcaaaaa@609C@

Alternatively, when the total case count C is assumed to conform to a compound Poisson process model, substitution of the earlier expression for Vâr(R) into (1) provides the following adjustment to interval (1a) to account for multiple-case incidents:

(exp⁡{ln⁡(R)−1.96×∑k=1NCk2C2},  exp⁡{ln⁡(R)+1.96×∑k=1NCk2C2})     (1b)
 MathType@MTEF@5@5@+=feaafiart1ev1aaatCvAUfKttLearuWrP9MDH5MBPbIqV92AaeXatLxBI9gBaebbnrfifHhDYfgasaacH8akY=wiFfYdH8Gipec8Eeeu0xXdbba9frFj0=OqFfea0dXdd9vqai=hGuQ8kuc9pgc9s8qqaq=dirpe0xb9q8qiLsFr0=vr0=vr0dc8meaabaqaciaacaGaaeqabaqabeGadaaakeaadaqadaqaaiGbcwgaLjabcIha4jabcchaWnaacmqabaGagiiBaWMaeiOBa4MaeiikaGIaeeOuaiLaeiykaKIaeyOeI0IaeGymaeJaeiOla4IaeGyoaKJaeGOnayJaey41aq7aaOaaaeaadaWcaaqaamaaqadabaGaee4qam0aa0baaSqaaiabbUgaRbqaaiabikdaYaaaaeaacqqGRbWAcqGH9aqpcqaIXaqmaeaacqqGobGta0GaeyyeIuoaaOqaaiabboeadnaaCaaaleqabaGaeGOmaidaaaaaaeqaaaGccaGL7bGaayzFaaGaeiilaWIaeeiiaaIaeeiiaaIagiyzauMaeiiEaGNaeiiCaa3aaiWabeaacyGGSbaBcqGGUbGBcqGGOaakcqqGsbGucqGGPaqkcqGHRaWkcqaIXaqmcqGGUaGlcqaI5aqocqaI2aGncqGHxdaTdaGcaaqaamaalaaabaWaaabmaeaacqqGdbWqdaqhaaWcbaGaee4AaSgabaGaeGOmaidaaaqaaiabbUgaRjabg2da9iabigdaXaqaaiabb6eaobqdcqGHris5aaGcbaGaee4qam0aaWbaaSqabeaacqaIYaGmaaaaaaqabaaakiaawUhacaGL9baaaiaawIcacaGLPaaacaWLjaGaaCzcamaabmaabaGaeGymaeJaeeOyaigacaGLOaGaayzkaaaaaa@7402@

where C_1_, C_2_, C_3_, ..., C_N _are the incident-specific case counts defined previously. By inspection of the square root terms in (1a) and (1b), it can be seen that the estimated variance of ln(R) is increased by the factor ∑k=1NCk2/C
 MathType@MTEF@5@5@+=feaafiart1ev1aaatCvAUfKttLearuWrP9MDH5MBPbIqV92AaeXatLxBI9gBaebbnrfifHhDYfgasaacH8akY=wiFfYdH8Gipec8Eeeu0xXdbba9frFj0=OqFfea0dXdd9vqai=hGuQ8kuc9pgc9s8qqaq=dirpe0xb9q8qiLsFr0=vr0=vr0dc8meaabaqaciaacaGaaeqabaqabeGadaaakeaadaaeWaqaaiabboeadnaaDaaaleaacqqGRbWAaeaacqaIYaGmaaaabaGaee4AaSMaeyypa0JaeGymaedabaGaeeOta4eaniabggHiLdGccqGGVaWlcqqGdbWqaaa@389F@ under the compound Poisson process model. When no multiple-case incidents appear in the data it follows that ∑k=1NCk2=∑k=1NCk≡C
 MathType@MTEF@5@5@+=feaafiart1ev1aaatCvAUfKttLearuWrP9MDH5MBPbIqV92AaeXatLxBI9gBaebbnrfifHhDYfgasaacH8akY=wiFfYdH8Gipec8Eeeu0xXdbba9frFj0=OqFfea0dXdd9vqai=hGuQ8kuc9pgc9s8qqaq=dirpe0xb9q8qiLsFr0=vr0=vr0dc8meaabaqaciaacaGaaeqabaqabeGadaaakeaadaaeWaqaaiabboeadnaaDaaaleaacqqGRbWAaeaacqaIYaGmaaaabaGaee4AaSMaeyypa0JaeGymaedabaGaeeOta4eaniabggHiLdGccqGH9aqpdaaeWaqaaiabboeadnaaBaaaleaacqqGRbWAaeqaaOGaeyyyIORaee4qamealeaacqqGRbWAcqGH9aqpcqaIXaqmaeaacqqGobGta0GaeyyeIuoaaaa@43A0@ (because C_k _= 1 for each incident covered), whereupon interval (1b) reduces to interval (1a) and the distinction between models becomes academic.

#### Example 1

This example briefly demonstrates the calculations required for intervals (1a) and (1b), referencing NVDRS summary data for the year 2004. These data (for 13 states) show that there were 25 homicide-suicide incidents involving homicide victims under 21 years of age. The left half of Table 1 provides a summary of the incident-specific homicide counts associated with these incidents. For example, the first summary line represents 19 separate incidents, each involving one homicide victim under 21 years old. The right half of the table shows the calculation of the sums appearing in intervals (1a) and (1b).

**Table 1:** NVDRS Summary Data and Calculations for a Rate Confidence Interval.

Incident Summary Data		Calculation of Sums	
Incident Count	Homicides <21 Years of Age	ΣC_k_	ΣC_k_^2^

19	1	19 × 1 = 19	19 × 1^2 ^= 19
6	2	6 × 2 = 12	6 × 2^2 ^= 24
		
25		31	43

The totals at the bottom of the right half of Table 1 are used to calculate the unadjusted and adjusted confidence interval estimates for the population-based rate. There were approximately 19.8 million persons under 21 years of age in the states covered by NVDRS for 2004.[18] Since the reporting period covered one year this translates to approximately 19.8 million person-years at risk for this age group, so the estimated rate per 100,000 person-years is:

R = (31/19.8M) × 100,000 ≈ 0.157.

Recalling the earlier shorthand notation C≡∑k=1NCk
 MathType@MTEF@5@5@+=feaafiart1ev1aaatCvAUfKttLearuWrP9MDH5MBPbIqV92AaeXatLxBI9gBaebbnrfifHhDYfgasaacH8akY=wiFfYdH8Gipec8Eeeu0xXdbba9frFj0=OqFfea0dXdd9vqai=hGuQ8kuc9pgc9s8qqaq=dirpe0xb9q8qiLsFr0=vr0=vr0dc8meaabaqaciaacaGaaeqabaqabeGadaaakeaacqqGdbWqcqGHHjIUdaaeWaqaaiabboeadnaaBaaaleaacqqGRbWAaeqaaaqaaiabbUgaRjabg2da9iabigdaXaqaaiabb6eaobqdcqGHris5aaaa@3885@, the unadjusted 95% confidence interval for the rate is obtained by substituting R ≈ 0.157 and C = 31 into (1a):

(exp⁡{ln⁡(0.157)−1.96×131},  exp⁡{ln⁡(0.157)+1.96×131})
 MathType@MTEF@5@5@+=feaafiart1ev1aaatCvAUfKttLearuWrP9MDH5MBPbIqV92AaeXatLxBI9gBaebbnrfifHhDYfgasaacH8akY=wiFfYdH8Gipec8Eeeu0xXdbba9frFj0=OqFfea0dXdd9vqai=hGuQ8kuc9pgc9s8qqaq=dirpe0xb9q8qiLsFr0=vr0=vr0dc8meaabaqaciaacaGaaeqabaqabeGadaaakeaadaqadaqaaiGbcwgaLjabcIha4jabcchaWnaacmqabaGagiiBaWMaeiOBa4MaeiikaGIaeGimaaJaeiOla4IaeGymaeJaeGynauJaeG4naCJaeiykaKIaeyOeI0IaeGymaeJaeiOla4IaeGyoaKJaeGOnayJaey41aq7aaOaaaeaadaWcaaqaaiabigdaXaqaaiabiodaZiabigdaXaaaaSqabaaakiaawUhacaGL9baacqGGSaalcqqGGaaicqqGGaaicyGGLbqzcqGG4baEcqGGWbaCdaGadeqaaiGbcYgaSjabc6gaUjabcIcaOiabicdaWiabc6caUiabigdaXiabiwda1iabiEda3iabcMcaPiabgUcaRiabigdaXiabc6caUiabiMda5iabiAda2iabgEna0oaakaaabaWaaSaaaeaacqaIXaqmaeaacqaIZaWmcqaIXaqmaaaaleqaaaGccaGL7bGaayzFaaaacaGLOaGaayzkaaaaaa@6376@

resulting in the interval estimate (0.110, 0.223).

The 95% confidence interval adjusted for multiple-case incidents is obtained by substituting the values R ≈ 0.157, C = 31, and ∑k=1NCk2
 MathType@MTEF@5@5@+=feaafiart1ev1aaatCvAUfKttLearuWrP9MDH5MBPbIqV92AaeXatLxBI9gBaebbnrfifHhDYfgasaacH8akY=wiFfYdH8Gipec8Eeeu0xXdbba9frFj0=OqFfea0dXdd9vqai=hGuQ8kuc9pgc9s8qqaq=dirpe0xb9q8qiLsFr0=vr0=vr0dc8meaabaqaciaacaGaaeqabaqabeGadaaakeaadaaeWaqaaiabboeadnaaDaaaleaacqqGRbWAaeaacqaIYaGmaaaabaGaee4AaSMaeyypa0JaeGymaedabaGaeeOta4eaniabggHiLdaaaa@36A2@ = 43 into (1b):

(exp⁡{ln⁡(0.157)−1.96×43312},  exp⁡{ln⁡(0.157)+1.96×43312}).
 MathType@MTEF@5@5@+=feaafiart1ev1aaatCvAUfKttLearuWrP9MDH5MBPbIqV92AaeXatLxBI9gBaebbnrfifHhDYfgasaacH8akY=wiFfYdH8Gipec8Eeeu0xXdbba9frFj0=OqFfea0dXdd9vqai=hGuQ8kuc9pgc9s8qqaq=dirpe0xb9q8qiLsFr0=vr0=vr0dc8meaabaqaciaacaGaaeqabaqabeGadaaakeaadaqadaqaaiGbcwgaLjabcIha4jabcchaWnaacmqabaGagiiBaWMaeiOBa4MaeiikaGIaeGimaaJaeiOla4IaeGymaeJaeGynauJaeG4naCJaeiykaKIaeyOeI0IaeGymaeJaeiOla4IaeGyoaKJaeGOnayJaey41aq7aaOaaaeaadaWcaaqaaiabisda0iabiodaZaqaaiabiodaZiabigdaXmaaCaaaleqabaGaeGOmaidaaaaaaeqaaaGccaGL7bGaayzFaaGaeiilaWIaeeiiaaIaeeiiaaIagiyzauMaeiiEaGNaeiiCaa3aaiWabeaacyGGSbaBcqGGUbGBcqGGOaakcqaIWaamcqGGUaGlcqaIXaqmcqaI1aqncqaI3aWncqGGPaqkcqGHRaWkcqaIXaqmcqGGUaGlcqaI5aqocqaI2aGncqGHxdaTdaGcaaqaamaalaaabaGaeGinaqJaeG4mamdabaGaeG4mamJaeGymaeZaaWbaaSqabeaacqaIYaGmaaaaaaqabaaakiaawUhacaGL9baaaiaawIcacaGLPaaacqGGUaGlaaa@6876@

The resulting interval estimate (0.104, 0.238) is approximately 19% wider than the unadjusted interval estimate.

#### Example 2

The coverage properties of the unadjusted and adjusted confidence intervals (1a) and (1b) were compared using stochastic simulation. Assuming that the Poisson distribution is an appropriate model for incident counts, the relevant simulation inputs are the underlying mean incident count λP and the underlying distribution of cases within incidents. The ranges of the simulation input values were selected in part to cover the observed values from Example 1.

The underlying mean incident count λP can be varied either through λ (the incident occurrence rate per person-year) or through P (person-years at risk) and for purposes of simulation the choice of which to vary is arbitrary. Therefore, P was held constant at 20 million person-years while λ was varied across the values 0.050 × 10^-5^, 0.125 × 10^-5^, 0.250 × 10^-5^, and 0.500 × 10^-5^. These values correspond to underlying mean incident counts of λP = 10, 25, 50, 100 for the hypothetical period of observation.

Each value of the incident occurrence rate λ was considered in combination with five different within-incident case count distributions. The within-incident case count distributions are denoted by quadruples (p_1_, p_2_, p_3_, p_4_) indicating the probabilities that an incident involves 1, 2, 3, or 4 cases of interest, respectively. The first within-incident distribution (0.76, 0.24, 0.00, 0.00) matches the observed distribution from Example 1 in which each incident involved one or two cases. The second within-incident distribution (0.95, 0.05, 0.00, 0.00) reflects a comparatively small fraction of incidents involving multiple cases. The remaining three distributions reflect a successively increasing fraction of incidents involving multiple cases as well as larger numbers of cases. Consistent with earlier notation, μ and σ^2 ^denote the mean and variance for each within-incident case count distribution.

The simulation was programmed using the Statistical Analysis System (SAS).[19] One-hundred thousand simulation replicates were generated for each combination of simulation inputs. Each replicate involved simulation of an incident count according to a Poisson distribution having the indicated mean λP, followed by simulation of incident-specific case counts according to the indicated discrete distribution. The 95% confidence intervals (1a) and (1b) were calculated for each simulation replicate, and for each interval estimate it was noted whether the underlying case rate per 100,000 person-years (λ × μ × 100,000) fell between the interval endpoints. The relative frequencies with which intervals (1a) and (1b) covered the true case rate for the various combinations of simulation inputs are displayed in Tables 2 and 3, respectively.

**Table 2:** Estimated Coverage for Unadjusted (Poisson) 95% Confidence Interval (1a).

			Incident Occurrence Rate per 100,000 Person-years (λ × 10^5^)			
			
			0.050	0.125	0.250	0.500
Within-Incident Case Count Distribution						
			Relative Frequency of Coverage			
(p_1_, p_2_, p_3_, p_4_)	μ	σ^2^				

(0.76, 0.24, 0.00, 0.00)	1.24	0.1824	0.912	0.908	0.906	0.899
(0.95, 0.05, 0.00, 0.00)	1.05	0.0475	0.944	0.941	0.937	0.944
(0.85, 0.10, 0.05, 0.00)	1.20	0.2600	0.914	0.896	0.908	0.900
(0.80, 0.15, 0.03, 0.02)	1.27	0.3771	0.891	0.884	0.892	0.891
(0.70, 0.20, 0.07, 0.03)	1.43	0.5651	0.867	0.864	0.864	0.854

**Table 3:** Estimated Coverage for Adjusted (Compound Poisson) 95% Confidence Interval (1b).

			Incident Occurrence Rate per 100,000 Person-years (λ × 10^5^)			
			
			0.050	0.125	0.250	0.500
Within-Incident Case Count Distribution						
			Relative Frequency of Coverage			
(p_1_, p_2_, p_3_, p_4_)	μ	σ^2^				

(0.76, 0.24, 0.00, 0.00)	1.24	0.1824	0.948	0.953	0.950	0.950
(0.95, 0.05, 0.00, 0.00)	1.05	0.0475	0.958	0.950	0.952	0.951
(0.85, 0.10, 0.05, 0.00)	1.20	0.2600	0.955	0.947	0.949	0.949
(0.80, 0.15, 0.03, 0.02)	1.27	0.3771	0.945	0.948	0.949	0.949
(0.70, 0.20, 0.07, 0.03)	1.43	0.5651	0.942	0.948	0.948	0.948

The results in Table 2 indicate that as incidents involving multiple cases and larger numbers of cases become more frequent, coverage for the unadjusted interval (1a) drops well below the nominal 95% level. In the first line of the table (paralleling the NVDRS data from Example 1) coverage falls to about 90%. In the second line, where multiple-case incidents are less common, the difference between effective and nominal coverage is minimal. In the fifth line of the table, representing the most extreme departure from one case per incident, coverage is reduced to just over 85%. In contrast, the results in Table 3 show that coverage for adjusted interval (1b) conforms closely to the nominal 95% level under all of the simulation parameter combinations considered.

Further simulation results (not shown) indicate that the performance of interval (1b) is sensitive to extra-Poisson variability in the incident counts. Although more general models compensating for such extra-Poisson variability might be considered, the primary interest here is the effect of multiple-case incidents. Moreover, multiple-case incidents are unmistakable when represented in the data, whereas extra-Poisson variability in the incident counts may be more difficult to detect. The remaining presentation therefore continues to rely on the compound Poisson process model, which adequately addresses the issue of multiple-case incidents and results in tractable computational expressions.

### Confidence Intervals for Rate Ratios

The analysis is somewhat more complicated when considering rate ratios as opposed to individual rates. Letting R_S1 _and R_S2 _denote rate estimators for two demographic subgroups (for example, persons under 21 years of age and persons 21 years of age or older) the estimated rate ratio is defined in the usual way as RR ≡ R_S1_/R_S2_. Applying a logarithmic transformation to this ratio, the delta method [11-14] suggests the following general form for an approximate 95% confidence interval for the underlying rate ratio:

(exp⁡{ln⁡(RR)−1.96×Va^r(RS1)RS12+Va^r(RS2)RS22−2×Co^v(RS1,RS2)RS1×RS2},exp⁡{ln⁡(RR)+1.96×Va^r(RS1)RS12+Va^r(RS2)RS22−2×Co^v(RS1,RS2)RS1×RS2}).     (2)
 MathType@MTEF@5@5@+=feaafiart1ev1aaatCvAUfKttLearuWrP9MDH5MBPbIqV92AaeXatLxBI9gBaebbnrfifHhDYfgasaacH8akY=wiFfYdH8Gipec8Eeeu0xXdbba9frFj0=OqFfea0dXdd9vqai=hGuQ8kuc9pgc9s8qqaq=dirpe0xb9q8qiLsFr0=vr0=vr0dc8meaabaqaciaacaGaaeqabaqabeGadaaakeGacaaydaaDeuaabmqaceaaaeaadaqabaqaaiGbcwgaLjabcIha4jabcchaWnaacmqabaGagiiBaWMaeiOBa4MaeiikaGIaeeOuaiLaeeOuaiLaeiykaKIaeyOeI0IaeGymaeJaeiOla4IaeGyoaKJaeGOnayJaey41aq7aaOaaaeaadaWcaaqaaiabbAfawjqbbggaHzaajaGaeeOCaiNaeiikaGIaeeOuai1aaSbaaSqaaiabbofatjabigdaXaqabaGccqGGPaqkaeaacqqGsbGudaqhaaWcbaGaee4uamLaeGymaedabaGaeGOmaidaaaaakiabgUcaRmaalaaabaGaeeOvayLafeyyaeMbaKaacqqGYbGCcqGGOaakcqqGsbGudaWgaaWcbaGaee4uamLaeGOmaidabeaakiabcMcaPaqaaiabbkfasnaaDaaaleaacqqGtbWucqaIYaGmaeaacqaIYaGmaaaaaOGaeyOeI0IaeGOmaiJaey41aq7aaSaaaeaacqqGdbWqcuqGVbWBgaqcaiabbAha2jabcIcaOiabbkfasnaaBaaaleaacqqGtbWucqaIXaqmaeqaaOGaeiilaWIaeeOuai1aaSbaaSqaaiabbofatjabikdaYaqabaGccqGGPaqkaeaacqqGsbGudaWgaaWcbaGaee4uamLaeGymaedabeaakiabgEna0kabbkfasnaaBaaaleaacqqGtbWucqaIYaGmaeqaaaaaaeqaaaGccaGL7bGaayzFaaGaeiilaWcacaGLOaaaaeaadaqacaqaceaa0rGaaCzcaiGbcwgaLjabcIha4jabcchaWnaacmqabaGagiiBaWMaeiOBa4MaeiikaGIaeeOuaiLaeeOuaiLaeiykaKIaey4kaSIaeGymaeJaeiOla4IaeGyoaKJaeGOnayJaey41aq7aaOaaaeaadaWcaaqaaiabbAfawjqbbggaHzaajaGaeeOCaiNaeiikaGIaeeOuai1aaSbaaSqaaiabbofatjabigdaXaqabaGccqGGPaqkaeaacqqGsbGudaqhaaWcbaGaee4uamLaeGymaedabaGaeGOmaidaaaaakiabgUcaRmaalaaabaGaeeOvayLafeyyaeMbaKaacqqGYbGCcqGGOaakcqqGsbGudaWgaaWcbaGaee4uamLaeGOmaidabeaakiabcMcaPaqaaiabbkfasnaaDaaaleaacqqGtbWucqaIYaGmaeaacqaIYaGmaaaaaOGaeyOeI0IaeGOmaiJaey41aq7aaSaaaeaacqqGdbWqcuqGVbWBgaqcaiabbAha2jabcIcaOiabbkfasnaaBaaaleaacqqGtbWucqaIXaqmaeqaaOGaeiilaWIaeeOuai1aaSbaaSqaaiabbofatjabikdaYaqabaGccqGGPaqkaeaacqqGsbGudaWgaaWcbaGaee4uamLaeGymaedabeaakiabgEna0kabbkfasnaaBaaaleaacqqGtbWucqaIYaGmaeqaaaaaaeqaaaGccaGL7bGaayzFaaaacaGLPaaacqGGUaGlcaWLjaGaaCzcamaabmaabaGaeGOmaidacaGLOaGaayzkaaaaaaaa@CB95@

Let C_S1 _and C_S2 _denote the case counts used to calculate R_S1 _and R_S2_, respectively. These counts (and hence R_S1 _and R_S2_) are often considered independent, and the covariance term in (2) thus omitted. Assuming that these counts follow a simple Poisson distribution, interval (2) reduces to the more customary interval [10,16,20] for the underlying rate ratio:

(exp⁡{ln⁡(RR)−1.96×1CS1+1CS2},  exp⁡{ln⁡(RR)+1.96×1CS1+1CS2}).     (2a)
 MathType@MTEF@5@5@+=feaafiart1ev1aaatCvAUfKttLearuWrP9MDH5MBPbIqV92AaeXatLxBI9gBaebbnrfifHhDYfgasaacH8akY=wiFfYdH8Gipec8Eeeu0xXdbba9frFj0=OqFfea0dXdd9vqai=hGuQ8kuc9pgc9s8qqaq=dirpe0xb9q8qiLsFr0=vr0=vr0dc8meaabaqaciaacaGaaeqabaqabeGadaaakeaadaqadaqaaiGbcwgaLjabcIha4jabcchaWnaacmqabaGagiiBaWMaeiOBa4MaeiikaGIaeeOuaiLaeeOuaiLaeiykaKIaeyOeI0IaeGymaeJaeiOla4IaeGyoaKJaeGOnayJaey41aq7aaOaaaeaadaWcaaqaaiabigdaXaqaaiabboeadnaaBaaaleaacqqGtbWucqaIXaqmaeqaaaaakiabgUcaRmaalaaabaGaeGymaedabaGaee4qam0aaSbaaSqaaiabbofatjabikdaYaqabaaaaaqabaaakiaawUhacaGL9baacqGGSaalcqqGGaaicqqGGaaicyGGLbqzcqGG4baEcqGGWbaCdaGadeqaaiGbcYgaSjabc6gaUjabcIcaOiabbkfasjabbkfasjabcMcaPiabgUcaRiabigdaXiabc6caUiabiMda5iabiAda2iabgEna0oaakaaabaWaaSaaaeaacqaIXaqmaeaacqqGdbWqdaWgaaWcbaGaee4uamLaeGymaedabeaaaaGccqGHRaWkdaWcaaqaaiabigdaXaqaaiabboeadnaaBaaaleaacqqGtbWucqaIYaGmaeqaaaaaaeqaaaGccaGL7bGaayzFaaaacaGLOaGaayzkaaGaeiOla4IaaCzcaiaaxMaadaqadaqaaiabikdaYiabbggaHbGaayjkaiaawMcaaaaa@71F8@

The adjustment to interval (2a) to account for multiple-case incidents must address dependencies not only within the two subgroups, but also dependencies extending across the subgroups. The latter type of dependency occurs when some multiple-case incidents include cases in both subgroups, and in such instances the covariance term in interval (2) cannot simply be omitted. Let C_S1·1_, C_S1·2_, C_S1·3_, ..., C_S1·N _denote the incident-specific case counts (some possibly zero) for subgroup 1 (so CS1≡∑k=1NCS1⋅k
 MathType@MTEF@5@5@+=feaafiart1ev1aaatCvAUfKttLearuWrP9MDH5MBPbIqV92AaeXatLxBI9gBaebbnrfifHhDYfgasaacH8akY=wiFfYdH8Gipec8Eeeu0xXdbba9frFj0=OqFfea0dXdd9vqai=hGuQ8kuc9pgc9s8qqaq=dirpe0xb9q8qiLsFr0=vr0=vr0dc8meaabaqaciaacaGaaeqabaqabeGadaaakeaacqqGdbWqdaWgaaWcbaGaee4uamLaeGymaedabeaakiabggMi6oaaqadabaGaee4qam0aaSbaaSqaaiabbofatjabigdaXiabgwSixlabbUgaRbqabaaabaGaee4AaSMaeyypa0JaeGymaedabaGaeeOta4eaniabggHiLdaaaa@3F3F@). Similarly, let C_S2·1_, C_S2·2_, C_S2·3_, ..., C_S2·N _denote the incident-specific case counts for subgroup 2 (so CS2≡∑k=1NCS2⋅k
 MathType@MTEF@5@5@+=feaafiart1ev1aaatCvAUfKttLearuWrP9MDH5MBPbIqV92AaeXatLxBI9gBaebbnrfifHhDYfgasaacH8akY=wiFfYdH8Gipec8Eeeu0xXdbba9frFj0=OqFfea0dXdd9vqai=hGuQ8kuc9pgc9s8qqaq=dirpe0xb9q8qiLsFr0=vr0=vr0dc8meaabaqaciaacaGaaeqabaqabeGadaaakeaacqqGdbWqdaWgaaWcbaGaee4uamLaeeOmaidabeaakiabggMi6oaaqadabaGaee4qam0aaSbaaSqaaiabbofatjabbkdaYiabgwSixlabbUgaRbqabaaabaGaee4AaSMaeyypa0JaeGymaedabaGaeeOta4eaniabggHiLdaaaa@3F35@). With P_S1 _and P_S2 _denoting person-years at risk for the respective subgroups, an unbiased estimate of Cov(R_S1_, R_S2_) under the compound Poisson process model is given by ∑k=1N(CS1⋅k×CS2⋅k)
 MathType@MTEF@5@5@+=feaafiart1ev1aaatCvAUfKttLearuWrP9MDH5MBPbIqV92AaeXatLxBI9gBaebbnrfifHhDYfgasaacH8akY=wiFfYdH8Gipec8Eeeu0xXdbba9frFj0=OqFfea0dXdd9vqai=hGuQ8kuc9pgc9s8qqaq=dirpe0xb9q8qiLsFr0=vr0=vr0dc8meaabaqaciaacaGaaeqabaqabeGadaaakeaadaaeWaqaamaabmaabaGaee4qam0aaSbaaSqaaiabbofatjabigdaXiabgwSixlabbUgaRbqabaGccqGHxdaTcqqGdbWqdaWgaaWcbaGaee4uamLaeGOmaiJaeyyXICTaee4AaSgabeaaaOGaayjkaiaawMcaaaWcbaGaee4AaSMaeyypa0JaeGymaedabaGaeeOta4eaniabggHiLdaaaa@44D4@/(P_S1 _× P_S2_) × 100,000^2 ^(see Appendix B). Substituting the appropriate variance and covariance estimates into (2) and simplifying provides the adjustment to interval (2a) to reflect both within-group and cross-group dependencies:

(exp⁡{ln⁡(RR)−1.96×∑k=1NCS1⋅k2CS12+∑k=1NCS2⋅k2CS22−2×∑k=1N(CS1⋅k×CS2⋅k)CS1×CS2},exp⁡{ln⁡(RR)+1.96×∑k=1NCS1⋅k2CS12+∑k=1NCS2⋅k2CS22−2×∑k=1N(CS1⋅k×CS2⋅k)CS1×CS2}).     (2b)
 MathType@MTEF@5@5@+=feaafiart1ev1aaatCvAUfKttLearuWrP9MDH5MBPbIqV92AaeXatLxBI9gBaebbnrfifHhDYfgasaacH8akY=wiFfYdH8Gipec8Eeeu0xXdbba9frFj0=OqFfea0dXdd9vqai=hGuQ8kuc9pgc9s8qqaq=dirpe0xb9q8qiLsFr0=vr0=vr0dc8meaabaqaciaacaGaaeqabaqabeGadaaakeGacaaieaaDeuaabmqaceaaaeaadaqabaqaaiGbcwgaLjabcIha4jabcchaWnaacmqabaGagiiBaWMaeiOBa4MaeiikaGIaeeOuaiLaeeOuaiLaeiykaKIaeyOeI0IaeGymaeJaeiOla4IaeGyoaKJaeGOnayJaey41aq7aaOaaaeaadaWcaaqaamaaqadabaGaee4qam0aa0baaSqaaiabbofatjabigdaXiabgwSixlabbUgaRbqaaiabikdaYaaaaeaacqqGRbWAcqGH9aqpcqaIXaqmaeaacqqGobGta0GaeyyeIuoaaOqaaiabboeadnaaDaaaleaacqqGtbWucqaIXaqmaeaacqaIYaGmaaaaaOGaey4kaSYaaSaaaeaadaaeWaqaaiabboeadnaaDaaaleaacqqGtbWucqaIYaGmcqGHflY1cqqGRbWAaeaacqaIYaGmaaaabaGaee4AaSMaeyypa0JaeGymaedabaGaeeOta4eaniabggHiLdaakeaacqqGdbWqdaqhaaWcbaGaee4uamLaeeOmaidabaGaeGOmaidaaaaakiabgkHiTiabikdaYiabgEna0oaalaaabaWaaabmaeaadaqadaqaaiabboeadnaaBaaaleaacqqGtbWucqaIXaqmcqGHflY1cqqGRbWAaeqaaOGaey41aqRaee4qam0aaSbaaSqaaiabbofatjabikdaYiabgwSixlabbUgaRbqabaaakiaawIcacaGLPaaaaSqaaiabbUgaRjabg2da9iabigdaXaqaaiabb6eaobqdcqGHris5aaGcbaGaee4qam0aaSbaaSqaaiabbofatjabigdaXaqabaGccqGHxdaTcqqGdbWqdaWgaaWcbaGaee4uamLaeGOmaidabeaaaaaabeaaaOGaay5Eaiaaw2haaiabcYcaSaGaayjkaaaabaWaaeGaaeGabaaPeiaaxMaacyGGLbqzcqGG4baEcqGGWbaCdaGadeqaaiGbcYgaSjabc6gaUjabcIcaOiabbkfasjabbkfasjabcMcaPiabgUcaRiabigdaXiabc6caUiabiMda5iabiAda2iabgEna0oaakaaabaWaaSaaaeaadaaeWaqaaiabboeadnaaDaaaleaacqqGtbWucqaIXaqmcqGHflY1cqqGRbWAaeaacqaIYaGmaaaabaGaee4AaSMaeyypa0JaeGymaedabaGaeeOta4eaniabggHiLdaakeaacqqGdbWqdaqhaaWcbaGaee4uamLaeGymaedabaGaeGOmaidaaaaakiabgUcaRmaalaaabaWaaabmaeaacqqGdbWqdaqhaaWcbaGaee4uamLaeGOmaiJaeyyXICTaee4AaSgabaGaeGOmaidaaaqaaiabbUgaRjabg2da9iabigdaXaqaaiabb6eaobqdcqGHris5aaGcbaGaee4qam0aa0baaSqaaiabbofatjabbkdaYaqaaiabikdaYaaaaaGccqGHsislcqaIYaGmcqGHxdaTdaWcaaqaamaaqadabaWaaeWaaeaacqqGdbWqdaWgaaWcbaGaee4uamLaeGymaeJaeyyXICTaee4AaSgabeaakiabgEna0kabboeadnaaBaaaleaacqqGtbWucqaIYaGmcqGHflY1cqqGRbWAaeqaaaGccaGLOaGaayzkaaaaleaacqqGRbWAcqGH9aqpcqaIXaqmaeaacqqGobGta0GaeyyeIuoaaOqaaiabboeadnaaBaaaleaacqqGtbWucqaIXaqmaeqaaOGaey41aqRaee4qam0aaSbaaSqaaiabbofatjabikdaYaqabaaaaaqabaaakiaawUhacaGL9baaaiaawMcaaiabc6caUiaaxMaacaWLjaWaaeWaaeaacqaIYaGmcqqGIbGyaiaawIcacaGLPaaaaaaaaa@F62D@

As with the adjusted interval for a rate, when no multiple-case incidents are represented in the data it follows that ∑k=1NCS1⋅k2=CS1
 MathType@MTEF@5@5@+=feaafiart1ev1aaatCvAUfKttLearuWrP9MDH5MBPbIqV92AaeXatLxBI9gBaebbnrfifHhDYfgasaacH8akY=wiFfYdH8Gipec8Eeeu0xXdbba9frFj0=OqFfea0dXdd9vqai=hGuQ8kuc9pgc9s8qqaq=dirpe0xb9q8qiLsFr0=vr0=vr0dc8meaabaqaciaacaGaaeqabaqabeGadaaakeaadaaeWaqaaiabboeadnaaDaaaleaacqqGtbWucqaIXaqmcqGHflY1cqqGRbWAaeaacqaIYaGmaaaabaGaee4AaSMaeyypa0JaeGymaedabaGaeeOta4eaniabggHiLdGccqGH9aqpcqqGdbWqdaWgaaWcbaGaee4uamLaeGymaedabeaaaaa@3F6F@, ∑k=1NCS2⋅k2=CS2
 MathType@MTEF@5@5@+=feaafiart1ev1aaatCvAUfKttLearuWrP9MDH5MBPbIqV92AaeXatLxBI9gBaebbnrfifHhDYfgasaacH8akY=wiFfYdH8Gipec8Eeeu0xXdbba9frFj0=OqFfea0dXdd9vqai=hGuQ8kuc9pgc9s8qqaq=dirpe0xb9q8qiLsFr0=vr0=vr0dc8meaabaqaciaacaGaaeqabaqabeGadaaakeaadaaeWaqaaiabboeadnaaDaaaleaacqqGtbWucqqGYaGmcqGHflY1cqqGRbWAaeaacqaIYaGmaaaabaGaee4AaSMaeyypa0JaeGymaedabaGaeeOta4eaniabggHiLdGccqGH9aqpcqqGdbWqdaWgaaWcbaGaee4uamLaeeOmaidabeaaaaa@3F65@, all cross-products C_S1·k _× C_S2·k _are zero, and the adjusted interval (2b) reduces to the unadjusted interval (2a).

#### Example 3

The calculations required for intervals (2a) and (2b) are illustrated using NVDRS summary data for the year 2004. These data indicate a total of 144 incidents (13 states) involving homicide followed by suicide. Of these, 127 incidents involved a single homicide, 15 involved a double homicide, one involved a triple homicide, and one involved a quadruple homicide, for a total of 164 homicides.[21] Expanding on Example 1, Table 4 provides a summary of the incident-specific homicide counts associated with all 144 homicide-suicide incidents, with homicides classified into the two demographic subgroups described earlier (<21 years of age, 21+ years of age).

**Table 4:** NVDRS Summary Data and Calculations for a Rate Ratio Confidence Interval.

Incident Summary Data				Calculation of Sums				
Incident Count	Homicides in Incident	Age <21	Age 21+	ΣC_S1·k_	ΣC_S1·k_^2^	ΣC_S2·k_	ΣC_S2·k_^2^	ΣC_S1·k _× C_S2·k_

14	1	1	0	14	14			
113	1	0	1			113	113	
4	2	2	0	8	16			
5	2	1	1	5	5	5	5	5
6	2	0	2			12	24	
1	3	2	1	2	4	1	1	2
1	4	2	2	2	4	2	4	4
				
144				31	43	133	147	11

The left half of Table 4 contains summary data for the homicide-suicide incidents. The right half of the table shows the calculation of the sums appearing in intervals (2a) and (2b). For example, the third summary line represents four separate incidents, each with two homicides in the first age group and none in the second age group. Since C_S1·k _= 2 and C_S2·k _= 0 for each of the four incidents represented by this line, it adds 4 × 2 = 8 to the sum ∑k=1NCS1⋅k
 MathType@MTEF@5@5@+=feaafiart1ev1aaatCvAUfKttLearuWrP9MDH5MBPbIqV92AaeXatLxBI9gBaebbnrfifHhDYfgasaacH8akY=wiFfYdH8Gipec8Eeeu0xXdbba9frFj0=OqFfea0dXdd9vqai=hGuQ8kuc9pgc9s8qqaq=dirpe0xb9q8qiLsFr0=vr0=vr0dc8meaabaqaciaacaGaaeqabaqabeGadaaakeaadaaeWaqaaiabboeadnaaBaaaleaacqqGtbWucqaIXaqmcqGHflY1cqqGRbWAaeqaaaqaaiabbUgaRjabg2da9iabigdaXaqaaiabb6eaobqdcqGHris5aaaa@3A16@ and 4 × 2^2 ^= 16 to the sum ∑k=1NCS1⋅k2
 MathType@MTEF@5@5@+=feaafiart1ev1aaatCvAUfKttLearuWrP9MDH5MBPbIqV92AaeXatLxBI9gBaebbnrfifHhDYfgasaacH8akY=wiFfYdH8Gipec8Eeeu0xXdbba9frFj0=OqFfea0dXdd9vqai=hGuQ8kuc9pgc9s8qqaq=dirpe0xb9q8qiLsFr0=vr0=vr0dc8meaabaqaciaacaGaaeqabaqabeGadaaakeaadaaeWaqaaiabboeadnaaDaaaleaacqqGtbWucqaIXaqmcqGHflY1cqqGRbWAaeaacqaIYaGmaaaabaGaee4AaSMaeyypa0JaeGymaedabaGaeeOta4eaniabggHiLdaaaa@3B09@.

The totals at the bottom of the right half of Table 4 are used to calculate the unadjusted and adjusted confidence interval estimates for the rate ratio. In the states covered by NVDRS for 2004, the size of the population for the first age group was approximately 19.8 million, and approximately 48.9 million for the second age group.[18] Since the reporting period covered one year, these population figures approximate person-years at risk in the respective age groups, so the estimated rate ratio is:

RR ≡ R_S1_/R_S2 _= (31/19.8M)/(133/48.9M) = (31/133) × (48.9/19.8) ≈ 0.576.

Again recalling the shorthand notation CS1≡∑k=1NCS1⋅k
 MathType@MTEF@5@5@+=feaafiart1ev1aaatCvAUfKttLearuWrP9MDH5MBPbIqV92AaeXatLxBI9gBaebbnrfifHhDYfgasaacH8akY=wiFfYdH8Gipec8Eeeu0xXdbba9frFj0=OqFfea0dXdd9vqai=hGuQ8kuc9pgc9s8qqaq=dirpe0xb9q8qiLsFr0=vr0=vr0dc8meaabaqaciaacaGaaeqabaqabeGadaaakeaacqqGdbWqdaWgaaWcbaGaee4uamLaeGymaedabeaakiabggMi6oaaqadabaGaee4qam0aaSbaaSqaaiabbofatjabigdaXiabgwSixlabbUgaRbqabaaabaGaee4AaSMaeyypa0JaeGymaedabaGaeeOta4eaniabggHiLdaaaa@3F3F@ and CS2≡∑k=1NCS2⋅k
 MathType@MTEF@5@5@+=feaafiart1ev1aaatCvAUfKttLearuWrP9MDH5MBPbIqV92AaeXatLxBI9gBaebbnrfifHhDYfgasaacH8akY=wiFfYdH8Gipec8Eeeu0xXdbba9frFj0=OqFfea0dXdd9vqai=hGuQ8kuc9pgc9s8qqaq=dirpe0xb9q8qiLsFr0=vr0=vr0dc8meaabaqaciaacaGaaeqabaqabeGadaaakeaacqqGdbWqdaWgaaWcbaGaee4uamLaeeOmaidabeaakiabggMi6oaaqadabaGaee4qam0aaSbaaSqaaiabbofatjabbkdaYiabgwSixlabbUgaRbqabaaabaGaee4AaSMaeyypa0JaeGymaedabaGaeeOta4eaniabggHiLdaaaa@3F35@, the unadjusted 95% confidence interval for the rate ratio is obtained by substituting RR ≈ 0.576 and the values C_S1 _= 31 and C_S2 _= 133 into (2a):

(exp⁡{ln⁡(0.576)−1.96×131+1133},  exp⁡{ln⁡(0.576)+1.96×131+1133})
 MathType@MTEF@5@5@+=feaafiart1ev1aaatCvAUfKttLearuWrP9MDH5MBPbIqV92AaeXatLxBI9gBaebbnrfifHhDYfgasaacH8akY=wiFfYdH8Gipec8Eeeu0xXdbba9frFj0=OqFfea0dXdd9vqai=hGuQ8kuc9pgc9s8qqaq=dirpe0xb9q8qiLsFr0=vr0=vr0dc8meaabaqaciaacaGaaeqabaqabeGadaaakeaadaqadaqaaiGbcwgaLjabcIha4jabcchaWnaacmqabaGagiiBaWMaeiOBa4MaeiikaGIaeGimaaJaeiOla4IaeGynauJaeG4naCJaeGOnayJaeiykaKIaeyOeI0IaeGymaeJaeiOla4IaeGyoaKJaeGOnayJaey41aq7aaOaaaeaadaWcaaqaaiabigdaXaqaaiabiodaZiabigdaXaaacqGHRaWkdaWcaaqaaiabigdaXaqaaiabigdaXiabiodaZiabiodaZaaaaSqabaaakiaawUhacaGL9baacqGGSaalcqqGGaaicqqGGaaicyGGLbqzcqGG4baEcqGGWbaCdaGadeqaaiGbcYgaSjabc6gaUjabcIcaOiabicdaWiabc6caUiabiwda1iabiEda3iabiAda2iabcMcaPiabgUcaRiabigdaXiabc6caUiabiMda5iabiAda2iabgEna0oaakaaabaWaaSaaaeaacqaIXaqmaeaacqaIZaWmcqaIXaqmaaGaey4kaSYaaSaaaeaacqaIXaqmaeaacqaIXaqmcqaIZaWmcqaIZaWmaaaaleqaaaGccaGL7bGaayzFaaaacaGLOaGaayzkaaaaaa@6CFE@

resulting in the interval estimate (0.390, 0.852).

To calculate the adjusted 95% confidence interval, the values RR ≈ 0.576, C_S1 _= 31, ∑k=1NCS1⋅k2
 MathType@MTEF@5@5@+=feaafiart1ev1aaatCvAUfKttLearuWrP9MDH5MBPbIqV92AaeXatLxBI9gBaebbnrfifHhDYfgasaacH8akY=wiFfYdH8Gipec8Eeeu0xXdbba9frFj0=OqFfea0dXdd9vqai=hGuQ8kuc9pgc9s8qqaq=dirpe0xb9q8qiLsFr0=vr0=vr0dc8meaabaqaciaacaGaaeqabaqabeGadaaakeaadaaeWaqaaiabboeadnaaDaaaleaacqqGtbWucqaIXaqmcqGHflY1cqqGRbWAaeaacqaIYaGmaaaabaGaee4AaSMaeyypa0JaeGymaedabaGaeeOta4eaniabggHiLdaaaa@3B09@ = 43, C_S2 _= 133, ∑k=1NCS2⋅k2
 MathType@MTEF@5@5@+=feaafiart1ev1aaatCvAUfKttLearuWrP9MDH5MBPbIqV92AaeXatLxBI9gBaebbnrfifHhDYfgasaacH8akY=wiFfYdH8Gipec8Eeeu0xXdbba9frFj0=OqFfea0dXdd9vqai=hGuQ8kuc9pgc9s8qqaq=dirpe0xb9q8qiLsFr0=vr0=vr0dc8meaabaqaciaacaGaaeqabaqabeGadaaakeaadaaeWaqaaiabboeadnaaDaaaleaacqqGtbWucqaIYaGmcqGHflY1cqqGRbWAaeaacqaIYaGmaaaabaGaee4AaSMaeyypa0JaeGymaedabaGaeeOta4eaniabggHiLdaaaa@3B0B@ = 147, and ∑k=1N(CS1⋅k×CS2⋅k)
 MathType@MTEF@5@5@+=feaafiart1ev1aaatCvAUfKttLearuWrP9MDH5MBPbIqV92AaeXatLxBI9gBaebbnrfifHhDYfgasaacH8akY=wiFfYdH8Gipec8Eeeu0xXdbba9frFj0=OqFfea0dXdd9vqai=hGuQ8kuc9pgc9s8qqaq=dirpe0xb9q8qiLsFr0=vr0=vr0dc8meaabaqaciaacaGaaeqabaqabeGadaaakeaadaaeWaqaamaabmaabaGaee4qam0aaSbaaSqaaiabbofatjabigdaXiabgwSixlabbUgaRbqabaGccqGHxdaTcqqGdbWqdaWgaaWcbaGaee4uamLaeGOmaiJaeyyXICTaee4AaSgabeaaaOGaayjkaiaawMcaaaWcbaGaee4AaSMaeyypa0JaeGymaedabaGaeeOta4eaniabggHiLdaaaa@44D4@ = 11 are substituted into expression (2b):

(exp⁡{ln⁡(0.576)−1.96×43312+1471332−2×1131×133},exp⁡{ln⁡(0.576)+1.96×43312+1471332−2×1131×133})
 MathType@MTEF@5@5@+=feaafiart1ev1aaatCvAUfKttLearuWrP9MDH5MBPbIqV92AaeXatLxBI9gBaebbnrfifHhDYfgasaacH8akY=wiFfYdH8Gipec8Eeeu0xXdbba9frFj0=OqFfea0dXdd9vqai=hGuQ8kuc9pgc9s8qqaq=dirpe0xb9q8qiLsFr0=vr0=vr0dc8meaabaqaciaacaGaaeqabaqabeGadaaakeGacaaydaaDeuaabmqaceaaaeaadaqabaqaaiGbcwgaLjabcIha4jabcchaWnaacmqabaGagiiBaWMaeiOBa4MaeiikaGIaeGimaaJaeiOla4IaeGynauJaeG4naCJaeGOnayJaeiykaKIaeyOeI0IaeGymaeJaeiOla4IaeGyoaKJaeGOnayJaey41aq7aaOaaaeaadaWcaaqaaiabisda0iabiodaZaqaaiabiodaZiabigdaXmaaCaaaleqabaGaeGOmaidaaaaakiabgUcaRmaalaaabaGaeGymaeJaeGinaqJaeG4naCdabaGaeGymaeJaeG4mamJaeG4mamZaaWbaaSqabeaacqaIYaGmaaaaaOGaeyOeI0IaeGOmaiJaey41aq7aaSaaaeaacqaIXaqmcqaIXaqmaeaacqaIZaWmcqaIXaqmcqGHxdaTcqaIXaqmcqaIZaWmcqaIZaWmaaaaleqaaaGccaGL7bGaayzFaaGaeiilaWcacaGLOaaaaeaadaqacaqaceaaOxGaaCzcaiGbcwgaLjabcIha4jabcchaWnaacmqabaGagiiBaWMaeiOBa4MaeiikaGIaeGimaaJaeiOla4IaeGynauJaeG4naCJaeGOnayJaeiykaKIaey4kaSIaeGymaeJaeiOla4IaeGyoaKJaeGOnayJaey41aq7aaOaaaeaadaWcaaqaaiabisda0iabiodaZaqaaiabiodaZiabigdaXmaaCaaaleqabaGaeGOmaidaaaaakiabgUcaRmaalaaabaGaeGymaeJaeGinaqJaeG4naCdabaGaeGymaeJaeG4mamJaeG4mamZaaWbaaSqabeaacqaIYaGmaaaaaOGaeyOeI0IaeGOmaiJaey41aq7aaSaaaeaacqaIXaqmcqaIXaqmaeaacqaIZaWmcqaIXaqmcqGHxdaTcqaIXaqmcqaIZaWmcqaIZaWmaaaaleqaaaGccaGL7bGaayzFaaaacaGLPaaaaaaaaa@91E3@

resulting in the interval estimate (0.375, 0.884).

The adjusted interval estimate is approximately 10% wider than the unadjusted estimate. In this example, the influence of the increased variance estimates for the rate ratio components (the numerator and denominator rate estimates) is partially offset by the covariance term.

When cases associated with multiple-case incidents are concentrated mostly within subgroups the covariance term in adjusted interval (2b) will be relatively small and this interval will generally be wider than unadjusted interval (2a). For example, if the subgroups represent separate geographic regions, multiple-case incidents will rarely involve both subgroups and the covariance term will be negligible. Conversely, in situations where multiple-case incidents frequently involve both subgroups, the covariance term in (2b) offsets the influence of cases concentrated within subgroups (as in the previous computational example). In some instances the covariance term can dominate to the extent that the adjusted interval is narrower than the unadjusted interval.

The performance of intervals (2a) and (2b) under the types of conditions just described was evaluated using stochastic simulation, assuming various combinations of the incident occurrence rate, the underlying rate ratio, and the within-incident case count distribution (p_1_, p_2_, p_3_, p_4_). The initial set of simulations randomly assigned all cases associated with any given multiple-case incident to one of the two subgroups (according to probabilities consistent with the assumed rate ratio) thereby reducing the covariance term in interval (2b) to zero. Provided that the underlying mean incident count for each subgroup was not less than 10, the estimated coverage for adjusted interval (2b) was within 1% of the nominal level (95%) for all simulation inputs considered. By contrast, the estimated coverage for unadjusted interval (2a) dropped to about 85% when assuming the most extreme within-incident case count distribution (0.70, 0.20, 0.07, 0.03) from Example 2.

When modified to include multiple-case incidents simultaneously involving both subgroups, the simulations again indicated an effective coverage for adjusted interval (2b) close to the nominal level for the inputs considered. However, the cross-group dependencies introduced by this simple change also resulted in an adjusted interval with average width about the same as that of the unadjusted interval. Consequently, the estimated coverage of unadjusted interval (2a) was also close to the nominal level.

### Confidence Intervals for Age-Standardized Rates and Rate Ratios

The methods considered thus far pertain to *crude *rates (and ratios of crude rates) estimated using a single case count (numerator) and a single value for person-years at risk (denominator). The treatment of *age-standardized *rates and ratios of age-standardized rates follows from straightforward extensions of results already presented.

To illustrate the proposed extension for age-standardized rates, assume that there are M age groups into which the data are partitioned and denote the corresponding age-group rate estimators by R_G1_, R_G2_, R_G3_, ..., R_GM_. Let ω_G1_, ω_G2_, ω_G3_, ..., ω_GM _denote corresponding age-group population fractions (assumed fixed) in the referent (standard) population, such that $\sum_{\ell =1}^{\text{M}}\omega_{\text{G}\ell }=1$. Applying the direct method of standardization [14] the age-standardized rate estimator is given by:

Ra≡∑ℓ=1MωGℓ×RGℓ.
 MathType@MTEF@5@5@+=feaafiart1ev1aaatCvAUfKttLearuWrP9MDH5MBPbIqV92AaeXatLxBI9gBaebbnrfifHhDYfgasaacH8akY=wiFfYdH8Gipec8Eeeu0xXdbba9frFj0=OqFfea0dXdd9vqai=hGuQ8kuc9pgc9s8qqaq=dirpe0xb9q8qiLsFr0=vr0=vr0dc8meaabaqaciaacaGaaeqabaqabeGadaaakeaacqqGsbGudaWgaaWcbaGaeeyyaegabeaakiabggMi6oaaqadabaacciGae8xYdC3aaSbaaSqaaiabbEeahjabloriSbqabaGccqGHxdaTcqqGsbGudaWgaaWcbaGaee4raCKaeS4eHWgabeaaaeaacqWItecBcqGH9aqpcqaIXaqmaeaacqqGnbqta0GaeyyeIuoakiabc6caUaaa@4250@

The usual formula for the variance of a weighted sum provides the following expression for the estimated variance of R_a_:

Va^r(Ra)=∑ℓ=1MωGℓ2×Va^r(RGℓ)+2×∑ℓ=1M−1∑m=ℓ+1MωGℓ×ωGm×Co^v(RGℓ,RGm).(3)
 MathType@MTEF@5@5@+=feaafiart1ev1aaatCvAUfKttLearuWrP9MDH5MBPbIqV92AaeXatLxBI9gBaebbnrfifHhDYfgasaacH8akY=wiFfYdH8Gipec8Eeeu0xXdbba9frFj0=OqFfea0dXdd9vqai=hGuQ8kuc9pgc9s8qqaq=dirpe0xb9q8qiLsFr0=vr0=vr0dc8meaabaqaciaacaGaaeqabaqabeGadaaakeaacqqGwbGvcuqGHbqygaqcaiabbkhaYjabcIcaOiabbkfasnaaBaaaleaacqqGHbqyaeqaaOGaeiykaKIaeyypa0ZaaabmaeaaiiGacqWFjpWDdaqhaaWcbaGaee4raCKaeS4eHWgabaGaeGOmaidaaOGaey41aqRaeeOvayLafeyyaeMbaKaacqqGYbGCcqGGOaakcqqGsbGudaWgaaWcbaGaee4raCKaeS4eHWgabeaakiabcMcaPiabgUcaRiabikdaYiabgEna0oaaqadabaWaaabmaeaacqWFjpWDdaWgaaWcbaGaee4raCKaeS4eHWgabeaakiabgEna0kab=L8a3naaBaaaleaacqqGhbWrcqqGTbqBaeqaaOGaey41aqRaee4qamKafe4Ba8MbaKaacqqG2bGDcqGGOaakcqqGsbGudaWgaaWcbaGaee4raCKaeS4eHWgabeaakiabcYcaSiabbkfasnaaBaaaleaacqqGhbWrcqqGTbqBaeqaaOGaeiykaKcaleaacqqGTbqBcqGH9aqpcqWItecBcqGHRaWkcqaIXaqmaeaacqqGnbqta0GaeyyeIuoaaSqaaiabloriSjabg2da9iabigdaXaqaaiabb2eanjabgkHiTiabigdaXaqdcqGHris5aaWcbaGaeS4eHWMaeyypa0JaeGymaedabaGaeeyta0eaniabggHiLdGccqGGUaGlcaWLjaWaaeWaaeaacqaIZaWmaiaawIcacaGLPaaaaaa@8090@

Here, dependencies between cases within any given age group will affect the variance of the age-group rate estimator, while dependencies between cases in different age groups will result in nonzero covariances between the age-group rate estimators. The analog to interval (1) for age-standardized rates is given by:

(exp⁡{ln⁡(Ra)−1.96×Va^r(Ra)Ra2},  exp⁡{ln⁡(Ra)+1.96×Va^r(Ra)Ra2}).     (4)
 MathType@MTEF@5@5@+=feaafiart1ev1aaatCvAUfKttLearuWrP9MDH5MBPbIqV92AaeXatLxBI9gBaebbnrfifHhDYfgasaacH8akY=wiFfYdH8Gipec8Eeeu0xXdbba9frFj0=OqFfea0dXdd9vqai=hGuQ8kuc9pgc9s8qqaq=dirpe0xb9q8qiLsFr0=vr0=vr0dc8meaabaqaciaacaGaaeqabaqabeGadaaakeaadaqadaqaaiGbcwgaLjabcIha4jabcchaWnaacmqabaGagiiBaWMaeiOBa4MaeiikaGIaeeOuai1aaSbaaSqaaiabbggaHbqabaGccqGGPaqkcqGHsislcqaIXaqmcqGGUaGlcqaI5aqocqaI2aGncqGHxdaTdaGcaaqaamaalaaabaGaeeOvayLafeyyaeMbaKaacqqGYbGCcqGGOaakcqqGsbGudaWgaaWcbaGaeeyyaegabeaakiabcMcaPaqaaiabbkfasnaaDaaaleaacqqGHbqyaeaacqaIYaGmaaaaaaqabaaakiaawUhacaGL9baacqGGSaalcqqGGaaicqqGGaaicyGGLbqzcqGG4baEcqGGWbaCdaGadeqaaiGbcYgaSjabc6gaUjabcIcaOiabbkfasnaaBaaaleaacqqGHbqyaeqaaOGaeiykaKIaey4kaSIaeGymaeJaeiOla4IaeGyoaKJaeGOnayJaey41aq7aaOaaaeaadaWcaaqaaiabbAfawjqbbggaHzaajaGaeeOCaiNaeiikaGIaeeOuai1aaSbaaSqaaiabbggaHbqabaGccqGGPaqkaeaacqqGsbGudaqhaaWcbaGaeeyyaegabaGaeGOmaidaaaaaaeqaaaGccaGL7bGaayzFaaaacaGLOaGaayzkaaGaeiOla4IaaCzcaiaaxMaadaqadaqaaiabisda0aGaayjkaiaawMcaaaaa@7613@

Appendix equations (A.1) and (B.1) provide variance and covariance estimation formulas applicable to the age-group rate estimators (with rate scale per 100,000 person-years) assuming a compound Poisson process model. These can be substituted into (3) to obtain a computational formula for Vâr(R_a_), which when used in (4) provides an interval adjusting for multiple-case incidents.

When considering a ratio of age-standardized rate estimates for two subgroups, there are three potential types of dependency associated with multiple-case incidents: (i) between cases within the same subgroup and age group, (ii) between cases in different age groups within the same subgroup, and (iii) between cases in different subgroups. All three effects can be simultaneously illustrated by considering the ratio of age-standardized rates for males and females. A multiple-case incident may variously involve several males (or females) in the same age group; males (or females) in different age groups (dependency between case counts contributing to the same age-standardized rate estimate); or both males and females (dependency between case counts contributing to both numerator and denominator rate estimates).

Letting R_a·S1 _and R_a·S2 _denote the respective age-standardized rate estimators for two subgroups, the age-standardized rate ratio is estimated by RR_a _≡ R_a·S1_/R_a·S2_. The analog to interval (2) for age-standardized rate ratios is:

(exp⁡{ln⁡(RRa)−1.96×Va^r(Ra⋅S1)Ra⋅S12+Va^r(Ra⋅S2)Ra⋅S22−2×Co^v(Ra⋅S1,Ra⋅S2)Ra⋅S1×Ra⋅S2},exp⁡{ln⁡(RRa)+1.96×Va^r(Ra⋅S1)Ra⋅S12+Va^r(Ra⋅S2)Ra⋅S22−2×Co^v(Ra⋅S1,Ra⋅S2)Ra⋅S1×Ra⋅S2}).     (5)
 MathType@MTEF@5@5@+=feaafiart1ev1aaatCvAUfKttLearuWrP9MDH5MBPbIqV92AaeXatLxBI9gBaebbnrfifHhDYfgasaacH8akY=wiFfYdH8Gipec8Eeeu0xXdbba9frFj0=OqFfea0dXdd9vqai=hGuQ8kuc9pgc9s8qqaq=dirpe0xb9q8qiLsFr0=vr0=vr0dc8meaabaqaciaacaGaaeqabaqabeGadaaakeGacaaydaaDeuaabmqaceaaaeaadaqabaqaaiGbcwgaLjabcIha4jabcchaWnaacmqabaGagiiBaWMaeiOBa4MaeiikaGIaeeOuaiLaeeOuai1aaSbaaSqaaiabbggaHbqabaGccqGGPaqkcqGHsislcqaIXaqmcqGGUaGlcqaI5aqocqaI2aGncqGHxdaTdaGcaaqaamaalaaabaGaeeOvayLafeyyaeMbaKaacqqGYbGCcqGGOaakcqqGsbGudaWgaaWcbaGaeeyyaeMaeyyXICTaee4uamLaeGymaedabeaakiabcMcaPaqaaiabbkfasnaaDaaaleaacqqGHbqycqGHflY1cqqGtbWucqaIXaqmaeaacqaIYaGmaaaaaOGaey4kaSYaaSaaaeaacqqGwbGvcuqGHbqygaqcaiabbkhaYjabcIcaOiabbkfasnaaBaaaleaacqqGHbqycqGHflY1cqqGtbWucqaIYaGmaeqaaOGaeiykaKcabaGaeeOuai1aa0baaSqaaiabbggaHjabgwSixlabbofatjabikdaYaqaaiabikdaYaaaaaGccqGHsislcqaIYaGmcqGHxdaTdaWcaaqaaiabboeadjqbb+gaVzaajaGaeeODayNaeiikaGIaeeOuai1aaSbaaSqaaiabbggaHjabgwSixlabbofatjabigdaXaqabaGccqGGSaalcqqGsbGudaWgaaWcbaGaeeyyaeMaeyyXICTaee4uamLaeGOmaidabeaakiabcMcaPaqaaiabbkfasnaaBaaaleaacqqGHbqycqGHflY1cqqGtbWucqaIXaqmaeqaaOGaey41aqRaeeOuai1aaSbaaSqaaiabbggaHjabgwSixlabbofatjabikdaYaqabaaaaaqabaaakiaawUhacaGL9baacqGGSaalaiaawIcaaaqaamaabiaabiqaaWWacaWLjaGagiyzauMaeiiEaGNaeiiCaa3aaiWabeaacyGGSbaBcqGGUbGBcqGGOaakcqqGsbGucqqGsbGudaWgaaWcbaGaeeyyaegabeaakiabcMcaPiabgUcaRiabigdaXiabc6caUiabiMda5iabiAda2iabgEna0oaakaaabaWaaSaaaeaacqqGwbGvcuqGHbqygaqcaiabbkhaYjabcIcaOiabbkfasnaaBaaaleaacqqGHbqycqGHflY1cqqGtbWucqaIXaqmaeqaaOGaeiykaKcabaGaeeOuai1aa0baaSqaaiabbggaHjabgwSixlabbofatjabigdaXaqaaiabikdaYaaaaaGccqGHRaWkdaWcaaqaaiabbAfawjqbbggaHzaajaGaeeOCaiNaeiikaGIaeeOuai1aaSbaaSqaaiabbggaHjabgwSixlabbofatjabikdaYaqabaGccqGGPaqkaeaacqqGsbGudaqhaaWcbaGaeeyyaeMaeyyXICTaee4uamLaeGOmaidabaGaeGOmaidaaaaakiabgkHiTiabikdaYiabgEna0oaalaaabaGaee4qamKafe4Ba8MbaKaacqqG2bGDcqGGOaakcqqGsbGudaWgaaWcbaGaeeyyaeMaeyyXICTaee4uamLaeGymaedabeaakiabcYcaSiabbkfasnaaBaaaleaacqqGHbqycqGHflY1cqqGtbWucqaIYaGmaeqaaOGaeiykaKcabaGaeeOuai1aaSbaaSqaaiabbggaHjabgwSixlabbofatjabigdaXaqabaGccqGHxdaTcqqGsbGudaWgaaWcbaGaeeyyaeMaeyyXICTaee4uamLaeGOmaidabeaaaaaabeaaaOGaay5Eaiaaw2haaaGaayzkaaGaeiOla4IaaCzcaiaaxMaadaqadaqaaiabiwda1aGaayjkaiaawMcaaaaaaaa@0760@

Computational formulas for Vâr(R_a·S1_) and Vâr(R_a·S2_) under the compound Poisson process model can be obtained as described above for interval (4). Appendix equation (C.1) provides a computational formula for Côv(R_a·S1_, R_a·S2_) (with rate scale per 100,000 person-years) assuming the compound Poisson process model. These formulas can be substituted into (5) to obtain an interval adjusting for the effects of multiple-case incidents.

When there are no multiple-case incidents represented in the data, the covariance estimates in (3) vanish as does the term Côv(R_a·S1_, R_a·S2_) appearing in (5). Under such circumstances (4) and (5) reduce to intervals appropriate when case counts are assumed to follow a simple Poisson distribution and subgroups are assumed independent.[22]

Stochastic simulation was used to evaluate the coverage properties of adjusted intervals (4) and (5) when case counts conform to a compound Poisson process model. When the age distributions of the study groups of interest do not depart too greatly from that of the referent population, the simulation results suggest that coverage levels are comparable to those reported earlier for the adjusted confidence intervals for crude rates and rate ratios. In particular, estimated coverage was close to the nominal level provided that underlying mean subgroup incident counts were not less than 10.

### Assessment of Bias

It was noted at the outset that crude rate estimators are unbiased; by extension age-standardized rate estimators are also unbiased. Consequently, the coverage properties of adjusted intervals for crude and age-standardized rates depend on the appropriateness of the compound Poisson process model as well as the accuracy of the normal approximation implied when applying the delta method.

While the crude and age-standardized rate estimators are unbiased, the rate ratio estimators are only asymptotically unbiased. A supplementary assessment of finite-sample bias in the simulations described following Example 3 suggests that it is relatively small compared to the standard error of the rate ratio estimator, for the simulation inputs considered. In none of the simulations was the bias strong enough to cause the effective coverage of the adjusted (compound Poisson) interval to differ substantially from the nominal level.

## Conclusion

By referring to the compound Poisson process model in place of the simple Poisson model for case counts, confidence intervals for injury-related mortality rates and rate ratios can be adjusted to account for statistical dependencies associated with multiple-case incidents. The adjustments rely on closed-form computations and offer meaningful improvements in the accuracy of statistical statements. The adjusted interval estimators described in this paper have been programmed as general routines using SAS.[19]

When the data show any pattern of multiple-case incidents, the adjusted intervals for rates will be wider than their unadjusted counterparts. This does not hold for the adjusted intervals for rate ratios, however; different patterns in the data can variously widen or narrow these intervals relative to their unadjusted counterparts.

It is evident that in situations where multiple-case incidents are very infrequent and involve small numbers of cases when they do occur, there will be little difference between the statistical results obtained using the methods based on the compound Poisson process model and those based on the simple Poisson model. In the context of the NVDRS data, for example, when suicides are considered separately there is almost no distinction between suicide-related incident counts and suicide case counts (because multiple-suicide incidents are extremely infrequent). Conversely, there may be situations covered by other reporting systems where multiple-case incidents are more prominent and/or involve larger numbers of cases than in the examples considered in this paper. Simulations show that in such instances, the gaps between nominal and effective coverage probabilities for the unadjusted interval estimators become quite substantial.

## Appendices

### A. The Estimated Variance of a Total Case Count

Let N ~ Poisson(λP) denote the count of incidents and let C_1_, C_2_, C_3_, ..., C_N _denote the incident-specific case counts. That ∑k=1NCk2
 MathType@MTEF@5@5@+=feaafiart1ev1aaatCvAUfKttLearuWrP9MDH5MBPbIqV92AaeXatLxBI9gBaebbnrfifHhDYfgasaacH8akY=wiFfYdH8Gipec8Eeeu0xXdbba9frFj0=OqFfea0dXdd9vqai=hGuQ8kuc9pgc9s8qqaq=dirpe0xb9q8qiLsFr0=vr0=vr0dc8meaabaqaciaacaGaaeqabaqabeGadaaakeaadaaeWaqaaiabboeadnaaDaaaleaacqqGRbWAaeaacqaIYaGmaaaabaGaee4AaSMaeyypa0JaeGymaedabaGaeeOta4eaniabggHiLdaaaa@36A2@ is an unbiased estimator for the variance of the total case count C≡∑k=1NCk
 MathType@MTEF@5@5@+=feaafiart1ev1aaatCvAUfKttLearuWrP9MDH5MBPbIqV92AaeXatLxBI9gBaebbnrfifHhDYfgasaacH8akY=wiFfYdH8Gipec8Eeeu0xXdbba9frFj0=OqFfea0dXdd9vqai=hGuQ8kuc9pgc9s8qqaq=dirpe0xb9q8qiLsFr0=vr0=vr0dc8meaabaqaciaacaGaaeqabaqabeGadaaakeaacqqGdbWqcqGHHjIUdaaeWaqaaiabboeadnaaBaaaleaacqqGRbWAaeqaaaqaaiabbUgaRjabg2da9iabigdaXaqaaiabb6eaobqdcqGHris5aaaa@3885@ under a compound Poisson process model can be demonstrated using a basic conditioning argument. Employing the assumptions specified in the text (particularly the independence assumptions) it follows that:

E[∑k=1NCk2]=EN[E[∑k=1NCk2|N]]=EN[∑k=1NE[Ck2|N]]=EN[∑k=1NE[Ck2]]=EN[N×(μ2+σ2)]=λP×(μ2+σ2).
 MathType@MTEF@5@5@+=feaafiart1ev1aaatCvAUfKttLearuWrP9MDH5MBPbIqV92AaeXatLxBI9gBaebbnrfifHhDYfgasaacH8akY=wiFfYdH8Gipec8Eeeu0xXdbba9frFj0=OqFfea0dXdd9vqai=hGuQ8kuc9pgc9s8qqaq=dirpe0xb9q8qiLsFr0=vr0=vr0dc8meaabaqaciaacaGaaeqabaqabeGadaaakeaafaqadeqbbaaaaeaacqqGfbqrcqGGBbWwdaaeWaqaaiabboeadnaaDaaaleaacqqGRbWAaeaacqaIYaGmaaaabaGaee4AaSMaeyypa0JaeGymaedabaGaeeOta4eaniabggHiLdGccqGGDbqxcqGH9aqpcqqGfbqrdaWgaaWcbaGaeeOta4eabeaakiabcUfaBjabbweafjabcUfaBnaaqadabaGaee4qam0aa0baaSqaaiabbUgaRbqaaiabikdaYaaaaeaacqqGRbWAcqGH9aqpcqaIXaqmaeaacqqGobGta0GaeyyeIuoakiabcYha8jabb6eaojabc2faDjabc2faDbqaaiabg2da9iabbweafnaaBaaaleaacqqGobGtaeqaaOGaei4waS1aaabmaeaacqqGfbqrcqGGBbWwcqqGdbWqdaqhaaWcbaGaee4AaSgabaGaeGOmaidaaOGaeiiFaWNaeeOta4ealeaacqqGRbWAcqGH9aqpcqaIXaqmaeaacqqGobGta0GaeyyeIuoakiabc2faDjabc2faDbqaaiabg2da9iabbweafnaaBaaaleaacqqGobGtaeqaaOGaei4waS1aaabmaeaacqqGfbqrcqGGBbWwcqqGdbWqdaqhaaWcbaGaee4AaSgabaGaeGOmaidaaOGaeiyxa0faleaacqqGRbWAcqGH9aqpcqaIXaqmaeaacqqGobGta0GaeyyeIuoakiabc2faDbqaaiabg2da9iabbweafnaaBaaaleaacqqGobGtaeqaaOGaei4waSLaeeOta4Kaey41aqRaeiikaGccciGae8hVd02aaWbaaSqabeaacqaIYaGmaaGccqGHRaWkcqWFdpWCdaahaaWcbeqaaiabikdaYaaakiabcMcaPiabc2faDbqaaiabg2da9iab=T7aSjabbcfaqjabgEna0kabcIcaOiab=X7aTnaaCaaaleqabaGaeGOmaidaaOGaey4kaSIae83Wdm3aaWbaaSqabeaacqaIYaGmaaGccqGGPaqkcqGGUaGlaaaaaa@9AFC@

Because the last term in the sequence of equalities corresponds to the underlying variance of the total case count C under the compound Poisson model, it follows that Va^r(C)=∑k=1NCk2
 MathType@MTEF@5@5@+=feaafiart1ev1aaatCvAUfKttLearuWrP9MDH5MBPbIqV92AaeXatLxBI9gBaebbnrfifHhDYfgasaacH8akY=wiFfYdH8Gipec8Eeeu0xXdbba9frFj0=OqFfea0dXdd9vqai=hGuQ8kuc9pgc9s8qqaq=dirpe0xb9q8qiLsFr0=vr0=vr0dc8meaabaqaciaacaGaaeqabaqabeGadaaakeaacqqGwbGvcuqGHbqygaqcaiabbkhaYjabcIcaOiabboeadjabcMcaPiabg2da9maaqadabaGaee4qam0aa0baaSqaaiabbUgaRbqaaiabikdaYaaaaeaacqqGRbWAcqGH9aqpcqaIXaqmaeaacqqGobGta0GaeyyeIuoaaaa@3E5E@ is an unbiased estimator for Var(C). Since the estimated case rate (per 100,000 person-years) is given by R ≡ C/P × 100,000, an unbiased estimate of Var(R) is:

Va^r(R)=Va^r(C)/P2×100,0002=∑k=1NCk2/P2×100,0002.     (A.1)
 MathType@MTEF@5@5@+=feaafiart1ev1aaatCvAUfKttLearuWrP9MDH5MBPbIqV92AaeXatLxBI9gBaebbnrfifHhDYfgasaacH8akY=wiFfYdH8Gipec8Eeeu0xXdbba9frFj0=OqFfea0dXdd9vqai=hGuQ8kuc9pgc9s8qqaq=dirpe0xb9q8qiLsFr0=vr0=vr0dc8meaabaqaciaacaGaaeqabaqabeGadaaakeaacqqGwbGvcuqGHbqygaqcaiabbkhaYjabcIcaOiabbkfasjabcMcaPiabg2da9iabbAfawjqbbggaHzaajaGaeeOCaiNaeiikaGIaee4qamKaeiykaKIaei4la8Iaeeiuaa1aaWbaaSqabeaacqaIYaGmaaGccqGHxdaTcqaIXaqmcqaIWaamcqaIWaamcqGGSaalcqaIWaamcqaIWaamcqaIWaamdaahaaWcbeqaaiabikdaYaaakiabg2da9maaqadabaGaee4qam0aa0baaSqaaiabbUgaRbqaaiabikdaYaaakiabc+caViabbcfaqnaaCaaaleqabaGaeGOmaidaaOGaey41aqRaeGymaeJaeGimaaJaeGimaaJaeiilaWIaeGimaaJaeGimaaJaeGimaaZaaWbaaSqabeaacqaIYaGmaaaabaGaee4AaSMaeyypa0JaeGymaedabaGaeeOta4eaniabggHiLdGccqGGUaGlcaWLjaGaaCzcamaabmaabaGaeeyqaeKaeiOla4IaeGymaedacaGLOaGaayzkaaaaaa@66A8@

### B. The Covariance of Rate Estimators

Let C_S1 _denote the total case count for subgroup 1 and let C_S1·1_, C_S1·2_, C_S1·3_, ..., C_S1·N _denote the incident-specific case counts (some possibly zero) for subgroup 1 (so CS1≡∑k=1NCS1⋅k
 MathType@MTEF@5@5@+=feaafiart1ev1aaatCvAUfKttLearuWrP9MDH5MBPbIqV92AaeXatLxBI9gBaebbnrfifHhDYfgasaacH8akY=wiFfYdH8Gipec8Eeeu0xXdbba9frFj0=OqFfea0dXdd9vqai=hGuQ8kuc9pgc9s8qqaq=dirpe0xb9q8qiLsFr0=vr0=vr0dc8meaabaqaciaacaGaaeqabaqabeGadaaakeaacqqGdbWqdaWgaaWcbaGaee4uamLaeGymaedabeaakiabggMi6oaaqadabaGaee4qam0aaSbaaSqaaiabbofatjabigdaXiabgwSixlabbUgaRbqabaaabaGaee4AaSMaeyypa0JaeGymaedabaGaeeOta4eaniabggHiLdaaaa@3F3F@). Similarly, for subgroup 2 let C_S2 _denote the total case count and let C_S2·1_, C_S2·2_, C_S2·3_, ..., C_S2·N _denote the incident-specific case counts (so CS2≡∑k=1NCS2⋅k
 MathType@MTEF@5@5@+=feaafiart1ev1aaatCvAUfKttLearuWrP9MDH5MBPbIqV92AaeXatLxBI9gBaebbnrfifHhDYfgasaacH8akY=wiFfYdH8Gipec8Eeeu0xXdbba9frFj0=OqFfea0dXdd9vqai=hGuQ8kuc9pgc9s8qqaq=dirpe0xb9q8qiLsFr0=vr0=vr0dc8meaabaqaciaacaGaaeqabaqabeGadaaakeaacqqGdbWqdaWgaaWcbaGaee4uamLaeeOmaidabeaakiabggMi6oaaqadabaGaee4qam0aaSbaaSqaaiabbofatjabbkdaYiabgwSixlabbUgaRbqabaaabaGaee4AaSMaeyypa0JaeGymaedabaGaeeOta4eaniabggHiLdaaaa@3F35@). The usual formula for the variance of a sum:

Var(C_S1 _+ C_S2_) = Var(C_S1_) + Var(C_S2_) + 2 × Cov(C_S1_, C_S2_)

can be rearranged to get:

Cov(C_S1_, C_S2_) = (Var(C_S1 _+ C_S2_) - Var(C_S1_) - Var(C_S2_))/2.

The results of Appendix A provide unbiased estimators for all of the terms appearing on the right-hand side of the last equality. Substituting these unbiased estimators provides an unbiased estimate of the covariance:

Co^v(CS1,CS2)=(∑k=1N(CS1⋅k+CS2⋅k)2−∑k=1NCS1⋅k2−∑k=1NCS2⋅k2)/2=∑k=1N(CS1⋅k×CS2⋅k).
 MathType@MTEF@5@5@+=feaafiart1ev1aaatCvAUfKttLearuWrP9MDH5MBPbIqV92AaeXatLxBI9gBaebbnrfifHhDYfgasaacH8akY=wiFfYdH8Gipec8Eeeu0xXdbba9frFj0=OqFfea0dXdd9vqai=hGuQ8kuc9pgc9s8qqaq=dirpe0xb9q8qiLsFr0=vr0=vr0dc8meaabaqaciaacaGaaeqabaqabeGadaaakeaafaqadeGabaaabaGaee4qamKafe4Ba8MbaKaacqqG2bGDcqGGOaakcqqGdbWqdaWgaaWcbaGaee4uamLaeGymaedabeaakiabcYcaSiabboeadnaaBaaaleaacqqGtbWucqaIYaGmaeqaaOGaeiykaKIaeyypa0JaeiikaGYaaabmaeaacqGGOaakcqqGdbWqdaWgaaWcbaGaee4uamLaeGymaeJaeyyXICTaee4AaSgabeaakiabgUcaRiabboeadnaaBaaaleaacqqGtbWucqaIYaGmcqGHflY1cqqGRbWAaeqaaOGaeiykaKYaaWbaaSqabeaacqaIYaGmaaGccqGHsisldaaeWaqaaiabboeadnaaDaaaleaacqqGtbWucqaIXaqmcqGHflY1cqqGRbWAaeaacqaIYaGmaaaabaGaee4AaSMaeyypa0JaeGymaedabaGaeeOta4eaniabggHiLdGccqGHsisldaaeWaqaaiabboeadnaaDaaaleaacqqGtbWucqaIYaGmcqGHflY1cqqGRbWAaeaacqaIYaGmaaaabaGaee4AaSMaeyypa0JaeGymaedabaGaeeOta4eaniabggHiLdGccqGGPaqkcqGGVaWlcqaIYaGmaSqaaiabbUgaRjabg2da9iabigdaXaqaaiabb6eaobqdcqGHris5aaGcbaGaeyypa0ZaaabmaeaacqGGOaakcqqGdbWqdaWgaaWcbaGaee4uamLaeGymaeJaeyyXICTaee4AaSgabeaakiabgEna0kabboeadnaaBaaaleaacqqGtbWucqaIYaGmcqGHflY1cqqGRbWAaeqaaOGaeiykaKIaeiOla4caleaacqqGRbWAcqGH9aqpcqaIXaqmaeaacqqGobGta0GaeyyeIuoaaaaaaa@8FC8@

Let P_S1 _and P_S2 _denote the respective person-years at risk in subgroups 1 and 2. The corresponding rate estimators (per 100,000 person-years) are R_S1 _≡ C_S1_/P_S1 _× 100,000 and R_S2 _≡ C_S2_/P_S2 _× 100,000. It follows immediately that an unbiased estimate of Cov(R_S1_, R_S2_) under a compound Poisson process model is given by:

Co^v(RS1,RS2)=∑k=1N(CS1⋅k×CS2⋅k)/(PS1×PS2)×100,0002.     (B.1)
 MathType@MTEF@5@5@+=feaafiart1ev1aaatCvAUfKttLearuWrP9MDH5MBPbIqV92AaeXatLxBI9gBaebbnrfifHhDYfgasaacH8akY=wiFfYdH8Gipec8Eeeu0xXdbba9frFj0=OqFfea0dXdd9vqai=hGuQ8kuc9pgc9s8qqaq=dirpe0xb9q8qiLsFr0=vr0=vr0dc8meaabaqaciaacaGaaeqabaqabeGadaaakeaacqqGdbWqcuqGVbWBgaqcaiabbAha2jabcIcaOiabbkfasnaaBaaaleaacqqGtbWucqaIXaqmaeqaaOGaeiilaWIaeeOuai1aaSbaaSqaaiabbofatjabikdaYaqabaGccqGGPaqkcqGH9aqpdaaeWaqaaiabcIcaOiabboeadnaaBaaaleaacqqGtbWucqaIXaqmcqGHflY1cqqGRbWAaeqaaOGaey41aqRaee4qam0aaSbaaSqaaiabbofatjabikdaYiabgwSixlabbUgaRbqabaGccqGGPaqkcqGGVaWlcqGGOaakcqqGqbaudaWgaaWcbaGaee4uamLaeGymaedabeaakiabgEna0kabbcfaqnaaBaaaleaacqqGtbWucqaIYaGmaeqaaOGaeiykaKIaey41aqRaeGymaeJaeGimaaJaeGimaaJaeiilaWIaeGimaaJaeGimaaJaeGimaaZaaWbaaSqabeaacqaIYaGmaaaabaGaee4AaSMaeyypa0JaeGymaedabaGaeeOta4eaniabggHiLdGccqGGUaGlcaWLjaGaaCzcamaabmaabaGaeeOqaiKaeiOla4IaeGymaedacaGLOaGaayzkaaaaaa@6F68@

### C. The Covariance of Age-Standardized Rate Estimators

Consider age-standardized rate estimators R_a·S1 _and R_a·S2 _for two subgroups based on a partition of the data into M age groups. Let R_S1·G1_, R_S1·G2_, R_S1·G3_, ... R_S1·GM _denote the age-group rate estimators (per 100,000 person-years) for subgroup 1 and similarly let R_S2·G1_, R_S2·G2_, R_S2·G3_, ... R_S2·GM _denote the age-group rate estimators for subgroup 2. Referring to the (fixed) age-group population fractions ω_G1_, ω_G2_, ω_G3_, ..., ω_GM _defined in the text, the covariance of the age-standardized rate estimators is given by:

Cov(Ra⋅S1,Ra⋅S2)=Cov(∑ℓ=1MωGℓ×RS1⋅Gℓ,∑m=1MωGm×RS2⋅Gm)=∑ℓ=1M∑m=1MωGℓ×ωGm×Cov(RS1⋅Gℓ,RS2⋅Gm).
 MathType@MTEF@5@5@+=feaafiart1ev1aaatCvAUfKttLearuWrP9MDH5MBPbIqV92AaeXatLxBI9gBaebbnrfifHhDYfgasaacH8akY=wiFfYdH8Gipec8Eeeu0xXdbba9frFj0=OqFfea0dXdd9vqai=hGuQ8kuc9pgc9s8qqaq=dirpe0xb9q8qiLsFr0=vr0=vr0dc8meaabaqaciaacaGaaeqabaqabeGadaaakeaafaqadeGabaaabaGaee4qamKaee4Ba8MaeeODayNaeiikaGIaeeOuai1aaSbaaSqaaiabbggaHjabgwSixlabbofatjabigdaXaqabaGccqGGSaalcqqGsbGudaWgaaWcbaGaeeyyaeMaeyyXICTaee4uamLaeGOmaidabeaakiabcMcaPiabg2da9iabboeadjabb+gaVjabbAha2jabcIcaOmaaqadabaacciGae8xYdC3aaSbaaSqaaiabbEeahjabloriSbqabaGccqGHxdaTieaacqGFsbGudaWgaaWcbaGaee4uamLaeGymaeJaeyyXICTaee4raCKaeS4eHWgabeaakiabcYcaSmaaqadabaGae8xYdC3aaSbaaSqaaiabbEeahjabb2gaTbqabaaabaGaeeyBa0Maeyypa0JaeGymaedabaGaeeyta0eaniabggHiLdGccqGHxdaTcqqGsbGudaWgaaWcbaGaee4uamLaeGOmaiJaeyyXICTaee4raCKaeeyBa0gabeaakiabcMcaPaWcbaGaeS4eHWMaeyypa0JaeGymaedabaGaeeyta0eaniabggHiLdaakeaacqGH9aqpdaaeWaqaamaaqadabaGae8xYdC3aaSbaaSqaaiabbEeahjabloriSbqabaGccqGHxdaTcqWFjpWDdaWgaaWcbaGaee4raCKaeeyBa0gabeaaaeaacqqGTbqBcqGH9aqpcqaIXaqmaeaacqqGnbqta0GaeyyeIuoakiabgEna0kabboeadjabb+gaVjabbAha2jabcIcaOiabbkfasnaaBaaaleaacqqGtbWucqaIXaqmcqGHflY1cqqGhbWrcqWItecBaeqaaOGaeiilaWIaeeOuai1aaSbaaSqaaiabbofatjabikdaYiabgwSixlabbEeahjabb2gaTbqabaGccqGGPaqkcqGGUaGlaSqaaiabloriSjabg2da9iabigdaXaqaaiabb2eanbqdcqGHris5aaaaaaa@A52A@

An unbiased estimate of the last expression on the right-hand side follows from the results of Appendix B. Specifically, for age group ℓ in subgroup 1, let C_S1·Gℓ·1_, C_S1·Gℓ·2_, C_S1·Gℓ·3_, ..., C_S1·Gℓ·N _denote the incident-specific case counts (some possibly zero) and let P_S1·Gℓ _denote the person-years at risk. Similarly, for age group m in subgroup 2, let C_S2·Gm·1_, C_S2·Gm·2_, C_S2·Gm·3_, ... C_S2·Gm·N _denote the incident-specific case counts and let P_S2·Gm _denote the person-years at risk. From (B.1) it follows that an unbiased estimate for Cov(R_a·S1_, R_a·S2_) is:

Co^v(Ra⋅S1,Ra⋅S2)=∑ℓ=1M∑m=1MωGℓ×ωGm×∑k=1N(CS1⋅Gℓ⋅k×CS2⋅Gm⋅k)/(PS1⋅Gℓ×PS2⋅Gm)×100,0002.     (C.1)
 MathType@MTEF@5@5@+=feaafiart1ev1aaatCvAUfKttLearuWrP9MDH5MBPbIqV92AaeXatLxBI9gBaebbnrfifHhDYfgasaacH8akY=wiFfYdH8Gipec8Eeeu0xXdbba9frFj0=OqFfea0dXdd9vqai=hGuQ8kuc9pgc9s8qqaq=dirpe0xb9q8qiLsFr0=vr0=vr0dc8meaabaqaciaacaGaaeqabaqabeGadaaakeaafaqaaeGabaaabaGaee4qamKafe4Ba8MbaKaacqqG2bGDcqGGOaakcqqGsbGudaWgaaWcbaGaeeyyaeMaeyyXICTaee4uamLaeGymaedabeaakiabcYcaSiabbkfasnaaBaaaleaacqqGHbqycqGHflY1cqqGtbWucqaIYaGmaeqaaOGaeiykaKIaeyypa0dabiqaaWQacaWLjaWaaabmaeaadaaeWaqaaGGaciab=L8a3naaBaaaleaacqqGhbWrcqWItecBaeqaaOGaey41aqRae8xYdC3aaSbaaSqaaiabbEeahjabb2gaTbqabaGccqGHxdaTdaaeWaqaaiabcIcaOiabboeadnaaBaaaleaacqqGtbWucqaIXaqmcqGHflY1cqqGhbWrcqWItecBcqGHflY1cqqGRbWAaeqaaOGaey41aqRaee4qam0aaSbaaSqaaiabbofatjabikdaYiabgwSixlabbEeahjabb2gaTjabgwSixlabbUgaRbqabaGccqGGPaqkcqGGVaWlcqGGOaakcqqGqbaudaWgaaWcbaGaee4uamLaeGymaeJaeyyXICTaee4raCKaeS4eHWgabeaakiabgEna0kabbcfaqnaaBaaaleaacqqGtbWucqaIYaGmcqGHflY1cqqGhbWrcqqGTbqBaeqaaOGaeiykaKIaey41aqRaeGymaeJaeGimaaJaeGimaaJaeiilaWIaeGimaaJaeGimaaJaeGimaaZaaWbaaSqabeaacqaIYaGmaaaabaGaee4AaSMaeyypa0JaeGymaedabaGaeeOta4eaniabggHiLdaaleaacqqGTbqBcqGH9aqpcqaIXaqmaeaacqqGnbqta0GaeyyeIuoakiabc6caUiaaxMaacaWLjaWaaeWaaeaacqqGdbWqcqGGUaGlcqaIXaqmaiaawIcacaGLPaaaaSqaaiabloriSjabg2da9iabigdaXaqaaiabb2eanbqdcqGHris5aaaaaaa@A445@

## Competing interests

The author(s) declare that they have no competing interests.
